# The influence of various distractions prior to upper gastrointestinal endoscopy: a prospective randomized controlled study

**DOI:** 10.1186/s12876-018-0859-y

**Published:** 2018-08-29

**Authors:** Masahiro Sogabe, Toshiya Okahisa, Yuka Adachi, Masanori Takehara, Shinichi Hamada, Jun Okazaki, Yasuteru Fujino, Akira Fukuya, Kaizo Kagemoto, Akihiro Hirao, Koichi Okamoto, Masahiko Nakasono, Tetsuji Takayama

**Affiliations:** 1 Department of Gastroenterology and Oncology, Institute of Biomedical Sciences, Tokushima University Graduate School, 3-18-15 Kuramoto-cho, Tokushima city, Tokushima, 770-8503 Japan; 2 Department of Internal Medicine, Shikoku Central Hospital of the Mutual aid Association of Public School teachers, Shikokuchuo, Japan; 3 Department of Internal Medicine, Tsurugi Municipal Handa Hospital, Tsurugi, Japan

**Keywords:** Distraction, Vital signs, Autonomic nervous system, Esophagoscopy, Music, Image

## Abstract

**Background:**

Although many patients still have anxiety about upper gastrointestinal (GI) endoscopy, there have been few reports on the influence of distractions for a person who is going to undergo upper GI endoscopy soon. This study was a prospective randomized controlled study investigating the influence of distractions, such as auditive and visual distractions using subjective and objective assessments including autonomic nervous function prior to upper GI endoscopy.

**Methods:**

206 subjects who underwent upper GI endoscopy as regular health check-ups were divided randomly into 4 groups prior to upper GI endoscopy; group 1 (control group), group 2 (auditive group), group 3 (visual group), and group 4 (combination group). We measured vital signs, autonomic nervous function, profile of mood state (POMS), and the impression for upper GI endoscopy pre- and post-distraction in the 4 groups.

**Results:**

There was no significant difference in vital signs between 5 and 15 min after sitting in group 1, however, several vital signs in all distraction groups improved significantly after distraction (Pulse rate (P): *p* <  0.001 in group 4; blood pressure: *p* <  0.05 in group 2, 3, 4) and the rate of decrease in P and diastolic blood pressure was highest in group 4 (*p* <  0.001). Several scores of POMS and the impression for upper GI endoscopy post-distraction improved significantly compared to pre-distraction between distraction groups and the satisfaction for distraction was highest in group 4 (*p* <  0.01). Regarding autonomic nerve function, the low- frequency power/ high- frequency power ratio post-distraction was significantly lower than that pre-distraction in all distraction groups (*p* <  0.001).

**Conclusions:**

Although auditive distraction alone and visual distraction alone were effective, a combination distraction was more effective than any other distraction by subjective and objective assessments. These distractions, which were simple and safe, may play an assistive role in the stability of physical and psychological conditions prior to upper GI endoscopy.

**Trial registration:**

This trial was registered in the University Hospital Medical Information Network (UMIN) Clinical Trials Registry as UMIN000022801. Registered on 10 July 2016.

## Background

Upper gastrointestinal (GI) endoscopy has become an indispensable examination to discover upper GI lesion. However, many patients still have feelings of vulnerability, fear, and embarrassment regarding upper GI endoscopy [1, 2]. Strong anxiety before upper GI endoscopy may be a reason why some patients avoid undergoing upper GI endoscopy. Additionally, high levels of anxiety may induce displeasure and lead to incomplete procedures. Although the use of medication for sedation is known to reduce anxiety and pain in patients undergoing endoscopy examination or endoscopic procedures, sedation may increase the likelihood of complications such as hypotension and depression of respiration [3–5]. Therefore, various noninvasive interventions, such as listening to music, have been used to attempt to improve patients’ anxiety during endoscopic examination. Although there are several reports that have examined the effect of listening to music or watching images during various endoscopic procedures, the majority of these reports were concerned with decreasing the dose of sedation and improving tolerance for pain and anxiety [1, 6–8]. Additionally, there have been few reports on the influence of distractions for a subject who is going to undergo upper GI endoscopy soon using subjective and objective assessments including vital signs, autonomic nerve function, and psychological questionnaires. In this study, we performed a prospective randomized controlled trial to assess the influence of distractions, such as auditive and visual distractions, prior to upper GI endoscopy.

## Methods

### Subjects and study design

**Fig. 1:**
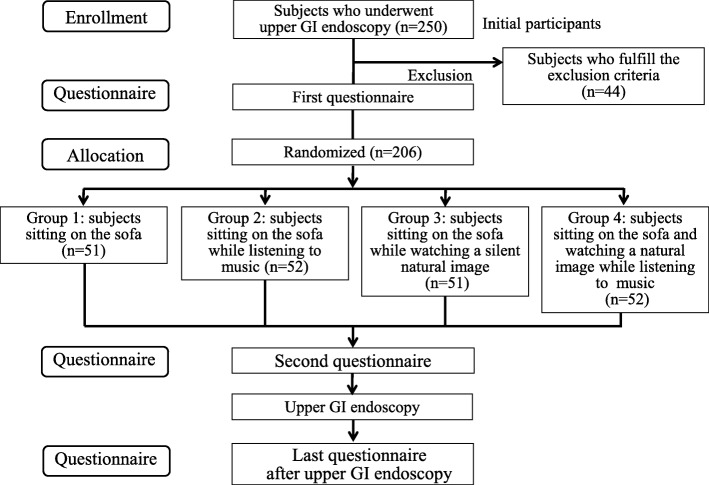
Flow chart of the enrollment and the procedures of this study. *GI* gastrointestinal

A flow chart of the enrollment and procedures of this study is shown in Fig. 1. Subjects included 250 individuals who underwent a regular health check-up including upper GI endoscopy at our hospital. The exclusion criteria were as follows: (1) attending a sedated upper GI endoscopy; (2) taking medicine; and (3) auditive and visual disability. Written informed consent was obtained from all subjects prior to their participation by a representative of this study. The endoscopy nurse who assisted at upper GI endoscopy performed the randomization divide into 4 groups by selecting sealed, opaque envelopes. We conducted a prospective randomized controlled trial from August 2016 to March 2017 at Shikoku Central Hospital of the Mutual Aid Association of Public School teachers. The study protocol was approved by the Ethics Committee in Shikoku Central Hospital of the Mutual Aid Association of Public School teachers and this study was performed in accordance with the Declaration of Helsinki. This trial was registered in the University Hospital Medical Information Network (UMIN) Clinical Trials Registry as UMIN000022801.

### Procedures of inspection

Participating subjects who were scheduled to receive upper GI endoscopy went to the endoscope floor after a more than 12 h fasting period, and were randomly divided into 4 groups. First, all subjects sat on a sofa and rested quietly for 5 min in a private room which was close to the endoscope room. Then, subjects in group 1 (control group) continued to sit on the sofa and rested quietly for 10 min prior to an upper GI endoscopy. Subjects in group 2 (auditive group) sat on the sofa while listening to music for 10 min. Subjects in group 3 (visual group) sat on the sofa while watching a silent natural image for 10 min. Subjects in group 4 (combination group) sat on the sofa and watched a natural image while listening to music for 10 min. The music used in this study was healing music such as country and classical based on the tone of a music box, and was chosen as good by 20 volunteers in the pre-meeting before the start of this study. The moving images used in this study were various natural images including a mountain, forest, river, waterfall, lake, and sunset. Music and natural images were supplied by using a wall-type Hi-vision liquid crystal television (TH-42AS650; Panasonic Corporation, Osaka Japan). And then, 3 endoscopy specialists who performed more than 180 upper GI endoscopy per month performed the endoscopic procedures after pharyngeal anesthesia with lidocaine pump spray (Xylocaine Pump Spray8%; AstraZeneca, Osaka, Japan) without any sedative agents.

### Measurement of cardiovascular and respiratory responses

Pulse rate (P), systolic blood pressure (SBP), diastolic blood pressure (DBP), and peripheral blood oxygen saturation (SpO_2_) were measured using a monitor unit (BSM-7100 Life Scope; NIHON KOHDEN CORPORATION, Tokyo, Japan). P and blood pressure were measured at the right upper arm. SpO_2_ was measured at the left finger. The parameters were determined at 5 and 15 min after sitting on the sofa. Changes in each parameter were calculated using the following equation: (values after sitting on the sofa for 5 min) - (values after sitting on the sofa for 15 min).

### Analysis of heart-rate variations

We measured heart rate variability (HRV) with a Heart Rhythm Scanner (HRV analysis system from Biocom Technologies, Ark Trading Pacific, Inc.) equipped with software that performs algorithms for short-term HRV analysis. Measurements were taken from subjects sitting on a sofa for 15 min in a private quiet room. A Biocom HRS − 08 Blue Tooth Wireless Pulse Wave Sensor, the photoplethysmography monitor used in this study, was clipped to the right earlobe to measure HRV for 15 min. We assessed autonomic nervous function while sitting on the sofa for 15 min by power spectral analysis (PSA). Data regarding the average R-R intervals for 5 min after sitting for 5 min and after sitting for 15 min were subjected to PSA using a software HRV analysis system. The amplitudes of the low-frequency range (LF, 0.04–0.15 Hz) and the high-frequency range (HF, 0.15–0.40 Hz) were analyzed by complex demodulation. The former was designated the low-frequency power (LF power), and the latter was designated the high-frequency power (HF power). The HF power represents the fluctuation in the heart rate caused by respiration, which is mediated by cardiac parasympathetic nervous activity [9, 10]. The ratio of LF power to HF power has been reported as an index of sympathetic nervous activity [9, 11, 12]. We converted HF power data to a logarithmic scale to make it possible to analyze with linear regression.

### Profile of mood states (POMS) questionnaire

A shortened Japanese version of POMS (POMS2), adapted from the original POMS standard version, was used in this study. POMS is known to be a self-report measure that allows for quick assessment of transient, fluctuating feelings and enduring affect states [13, 14]. The POMS2 is composed of 35 items rated on a scale from 0 to 4, namely 0) “not at all,” 1) “a little,” 2) “moderately,” 3) “quite a bit,” and 4) “extremely”. The checklist items are comprised of 8 subscale scores: anger-hostility (A-H), confusion-bewilderment (C-B), depression-dejection (D-D), fatigue-languid (F-I), tension-anxiety (T-A), total mood distress (TMD), vigor-vitality (V-V), and friendship (F). All subjects completed the POMS scale to measure psychological well-being at baseline conditions (immediately after sitting on the sofa) and 15 min after sitting on the sofa.

### Assessment of the impression for upper GI endoscopy

We investigated the impression for upper GI endoscopy at baseline conditions (immediately after sitting on the sofa) and 15 min after sitting on the sofa with distraction. We used questionnaires of the Visual Analog Scale (VAS) consisting of a 100-mm horizontal line that was scored from 0 (none) to 100 (strong) for the degree of strain, anxiety, and fear for upper GI endoscopy.

### Impression of distraction after upper GI endoscopy

After upper GI endoscopy examination, we investigated the impression of distraction using the questionnaire about the satisfaction for distraction and the willing for the use of distraction at next upper GI endoscopy examination. We used questionnaires of VAS consisting of a 100-mm horizontal line that was scored from 0 (none) to 100 (strong satisfaction or willing).

### Statistical analysis

We determined that the appropriate sample size for the randomized subjects was over 180 subjects. This was based on the requirement of a significant difference between 4 groups with a significance level of 0.05, power of 80%, and, effect size of 0.25. Additionally, we estimated that the required number of subject who receive upper GI endoscopy was over 250 in consideration of the exclusion criteria. This was based on the assumption of 25% that was the rate of subjects who fill exclusion criteria by referring to our previous prospective randomized trial on endoscopy. Baseline data, including subject characteristics such as age, number of upper GI endoscopy, POMS, VAS, P, blood pressure, and SpO_2_, are expressed as the means ± standard deviation (SD). Also, parameters of autonomic nervous function are expressed as the means ± standard error of the mean (SEM). All statistical differences at a *p* value less of than 0.05 were considered significant. The χ^2^-test or the Mann-Whitney U-test was used to compare between 2 groups or pre- and post-distraction in same group. The Kruskal Wallis-test or m × n χ^2^-test was used to compare among 3 or 4 groups. If the Kruskal Wallis-test show the difference in the groups, post-hoc pairwise comparisons were made using the Mann-Whitney U test with Bonferroni correction. All analyses were performed using Med Calc Software (Broekstraat, Mariakerke, Belgium).

## Results

### Characteristics of subjects

Subject characteristics are shown in Table 1. The proportion of males and females were 58.3% and 41.7%, respectively. The mean age was 51.8 ± 6.6 years. The mean number of upper GI endoscopy was 4.0 ± 3.4. The mean of P, SBP, DBP, and SpO_2_ was 65.2 ± 9.2 /min, 124.0 ± 15.9 mmHg, 80.1 ± 12.0 mmHg, and 98.2 ± 1.3%, respectively.

**Table 1:** Subject characteristics

Number	206
Gender (male/female) (% male)	120/86 (58.3%)
Age (years)	51.8 ± 6.6 (34–62)
Number of upper GI endoscopy experience	4.0 ± 3.4 (0–20)
P (/min)	65.2 ± 9.2 (42–93)
SBP (mmHg)	124.0 ± 15.9 (91–178)
DBP (mmHg)	80.1 ± 12.0 (46–116)
SpO_2_ (%)	98.2 ± 1.3 (93–100)

*DBP* diastolic blood pressure; *GI* gastrointestinal; *P* pulse; *SBP* systolic blood pressure; *SpO*_*2*_ peripheral blood oxygen saturation

Data represent the means ± standard deviation (SD)

### Comparison of baseline characteristics among the four groups

A comparison of the baseline characteristics among the 4 groups is shown in Table 2. There was no significant difference in gender, age, the frequency, and the duration of upper GI endoscopy among the 4 groups. There was no significant difference in the baseline score of POMS and the impression for upper GI endoscopy among the 4 groups.

**Table 2:** Comparison of baseline characteristics among the four groups

	Group 1	Group 2	Group 3	Group 4	*p*-value
Number	51	52	51	52	NS
Gender (male/female)	32/19	33/19	29/22	26/26	NS
Age (years)	52.4 ± 6.5	52.0 ± 6.3	50.7 ± 7.5	52.1 ± 6.2	NS
Number of upper GI endoscopy experience	3.9 ± 3.8	4.8 ± 4.1	3.4 ± 2.7	3.7 ± 2.9	NS
Duration of upper GI endoscopy (seconds)	358 ± 104	377 ± 84	371 ± 121	361 ± 86	NS
First Score of POMS					
(Score of negative mood)					
A-H	46.9 ± 8.4	46.7 ± 7.1	47.8 ± 7.0	45.2 ± 7.7	NS
C-B	48.1 ± 8.6	49.8 ± 8.4	50.4 ± 7.1	47.5 ± 8.4	NS
D-D	48.7 ± 7.9	50.1 ± 8.6	49.3 ± 6.8	48.4 ± 6.7	NS
F-I	45.7 ± 10.0	46.6 ± 7.2	46.3 ± 7.3	44.2 ± 8.2	NS
T-A	53.0 ± 10.9	51.1 ± 9.0	54.7 ± 9.4	50.3 ± 10.4	NS
TMD	47.4 ± 9.2	47.8 ± 7.9	48.3 ± 7.0	45.1 ± 7.7	NS
(Score of positive mood)					
V-V	55.3 ± 10.8	55.9 ± 9.1	56.2 ± 9.1	53.0 ± 10.3	NS
F	57.2 ± 9.6	59.3 ± 8.5	59.8 ± 8.9	60.2 ± 9.3	NS
VAS of first impression for upper GI endoscopy					
Strain	45.2 ± 27.7	42.0 ± 25.3	53.9 ± 28.9	41.7 ± 28.7	NS
Anxiety	34.4 ± 28.2	39.3 ± 25.0	33.8 ± 24.1	31.3 ± 26.7	NS
Fear	22.3 ± 23.1	28.1 ± 24.4	26.8 ± 18.8	21.8 ± 21.9	NS

*A-H* anger-hostility, *C-B* confusion-bewilderment, *D-D* depression-dejection, *F* friendship, *F-I* fatigue-languid, *GI* gastrointestinal, *POMS* profile of mood states, *T-A* tension-anxiety, *TMD* total mood distress, *V-V* vigor-vitality, *VAS* visual analog scale

Data represent the means ± standard deviation (SD), and number for categorical variables. *P*-value is based on the m × n χ^2^ test or Kruskal Wallis-test. Significance is at the 5% level

### Influence of distraction on vital signs

**Fig. 2:**
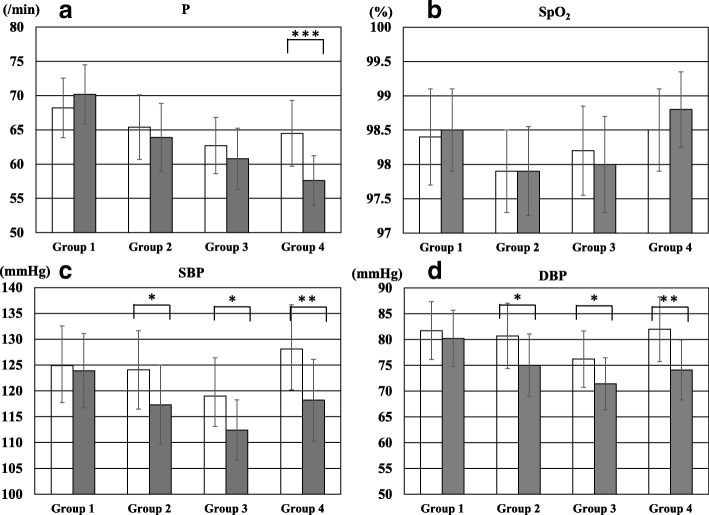
Comparison of vital signs between pre- and post-distractions in the 4 groups. **a** Comparison of P between pre- and post-distraction. **b** Comparison of SpO_2_ between pre- and post-distraction. **c** Comparison of SBP between pre- and post-distraction. **d** Comparison of DBP between pre- and post-distraction. The white bar indicates the value at pre-distraction. The gray bar indicates value at post-distraction. DBP, diastolic blood pressure; P, pulse; SBP, systolic blood pressure; SpO_2_, peripheral blood oxygen saturation; *p <  0.05; **p <  0.005; ***p <  0.001

The comparison of vital signs between pre- and post-distraction among the 4 groups is shown in Fig. 2. P post-distraction was significantly lower than that pre-distraction in group 4 (*p* <  0.001). SBP and DBP post-distraction was significantly lower than that pre-distraction in group 2, group 3, and group 4 (*p* <  0.05, *p* <  0.05, and *p* <  0.005). There was no significant difference in SpO_2_ between pre- and post-distraction among the 4 groups. A comparison of the rate of decrease in vital signs between pre- and post-distraction among the 4 groups is shown in Table 3. There was a significant difference in the rate of decrease in P, SBP, and DBP among the 4 groups on the Kruskal Wallis-test (< 0.001). In post-hoc pairwise comparisons, the rate of decrease in P and DBP in group 4 was significantly higher than that in other 3 groups.

**Table 3:** Comparison of the rate of decrease in vital sign between pre- and post-distraction among the four groups

Vital sign		Group				*P*-value
		Group 1	Group 2	Group 3	Group 4	
P (/min)	Mean	-1.98^a^	1.50^b^	1.90^b^	6.92^c^	< 0.001
	SEM	0.61	0.37	0.35	0.54	
SpO_2_ (%)	Mean	−0.14^a^	−0.04^a^	0.12^a^	-0.25^a^	NS
	SEM	0.12	0.15	0.09	0.15	
SBP (mmHg)	Mean	1.00^a^	6.77^b^	6.55^bc^	9.92^c^	< 0.001
	SEM	0.92	1.23	0.98	1.31	
DBP (mmHg)	Mean	1.51^a^	5.71^b^	4.77^b^	7.83^c^	< 0.001
	SEM	0.59	1.24	0.77	0.81	

*DBP*, diastolic blood pressure; *P*, pulse; *SBP*, systolic blood pressure; *SEM*, standard error of the mean; *SpO*_*2*_, peripheral blood oxygen saturation

*P*-value is based on the Kruskal Wallis-test. Significance is at the 5% level. Post hoc pairwise comparison were conducted by Mann-Whitney U test with the Bonferroni correction; different letters indicated a significant difference at the 0.00833 (0.05/6) level

### Influence of distraction on autonomic nerve function

**Fig. 3:**
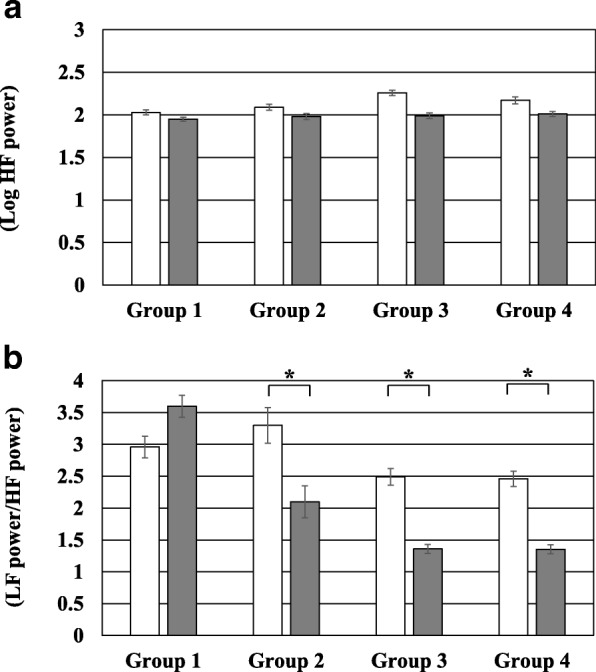
Comparison of autonomic nerve function between pre- and post-distraction among the 4 groups. **a** Comparison of Log HF power between pre- and post-distraction among the 4 groups. **b** Comparison of LF power/ HF power ratio between pre- and post-distraction among the 4 groups. The white bar indicates the value at pre-distraction. The gray bar indicates value at post-distraction. HF, high frequency; *LF* low frequency; *p <  0.001

The comparison of autonomic nerve function between pre- and post-distraction among the 4 groups is shown in Fig. 3. There was no significant difference in Log HF power between pre- and post-distraction among the 4 groups. The LF power/ HF power ratio post-distraction was significantly lower than that pre-distraction in group 2, group 3, and group 4 (*p* <  0.001).

### Influence of distraction on POMS and the impression for upper GI endoscopy

The comparison of POMS and the impression for upper GI endoscopy between pre- and post-distraction among the 3 groups is shown in Table 4. The score of A-H, F-I, T-A, and TMD post-distraction was significantly lower than that pre-distraction in group 2 (*p* <  0.05, *p* <  0.05, *p* <  0.01, and *p* <  0.05). The score of C-B, D-D, F-I, T-A, and TMD post-distraction was significantly lower than that pre-distraction in group 3 (*p* <  0.05, *p* <  0.01, *p* <  0.05, *p* <  0.01, and *p* <  0.01). The score of C-B, D-D, F-I, T-A, and TMD post-distraction was significantly lower than that pre-distraction in group 4 (*p* <  0.05, *p* <  0.01, *p* <  0.01, *p* <  0.01, and *p* <  0.01). However, there was no significant difference in the score of positive mood between pre- and post-distraction among group 2, group 3, and group 4.

**Table 4:** Comparison of POMS and the impression for upper GI endoscopy between pre- and post-distraction among the three groups

	POMS and Impression for upper GI endoscopy	Pre-distraction	Post-distraction	*P*-value
Group 2	(POMS: Score of negative mood)			
	A-H	46.7 ± 7.1	44.0 ± 6.8	< 0.05
	C-B	49.8 ± 8.4	47.4 ± 8.0	NS
	D-D	50.1 ± 8.6	47.5 ± 7.4	NS
	F-I	46.6 ± 7.2	43.4 ± 7.1	< 0.05
	T-A	51.1 ± 9.0	45.9 ± 8.9	< 0.01
	TMD	47.8 ± 7.9	44.0 ± 7.8	< 0.05
	(POMS: Score of positive mood)			
	V-V	55.9 ± 9.1	56.2 ± 10.0	NS
	F	59.3 ± 8.5	59.5 ± 10.3	NS
	(Impression for endoscopy)			
	Strain	42.0 ± 25.3	32.9 ± 23.2	NS
	Anxiety	39.3 ± 25.0	27.9 ± 23.5	< 0.05
	Fear	28.1 ± 24.4	19.5 ± 21.2	NS
Group 3	(POMS: Score of negative mood)			
	A-H	47.8 ± 7.0	45.7 ± 7.5	NS
	C-B	50.4 ± 7.1	47.3 ± 7.6	< 0.05
	D-D	49.3 ± 6.8	46.4 ± 6.4	< 0.01
	F-I	46.3 ± 7.3	43.3 ± 6.8	< 0.05
	T-A	54.7 ± 9.4	48.0 ± 8.1	< 0.01
	TMD	48.3 ± 7.0	44.3 ± 6.8	< 0.01
	(POMS: Score of positive mood)			
	V-V	56.2 ± 9.1	55.3 ± 11.2	NS
	F	59.8 ± 8.9	59.4 ± 10.9	NS
	(Impression for endoscopy)			
	Strain	53.9 ± 28.9	38.8 ± 21.5	< 0.01
	Anxiety	33.8 ± 24.1	31.3 ± 23.8	NS
	Fear	26.8 ± 18.8	27.0 ± 26.4	NS
Group 4	(POMS: Score of negative mood)			
	A-H	45.2 ± 7.7	41.9 ± 6.5	NS
	C-B	47.5 ± 8.4	44.1 ± 7.0	< 0.05
	D-D	48.4 ± 6.7	44.7 ± 6.1	< 0.01
	F-I	44.2 ± 8.2	39.0 ± 6.1	< 0.01
	T-A	50.3 ± 10.4	42.6 ± 8.8	< 0.01
	TMD	45.1 ± 7.7	41.3 ± 6.6	< 0.01
	(POMS: Score of positive mood)			
	V-V	53.0 ± 10.3	53.2 ± 12.1	NS
	F	60.2 ± 9.3	60.2 ± 10.5	NS
	(Impression for endoscopy)			
	Strain	41.7 ± 28.7	21.5 ± 23.5	< 0.001
	Anxiety	31.3 ± 26.7	19.2 ± 21.9	< 0.05
	Fear	21.8 ± 21.9	13.4 ± 19.4	< 0.05

*A-H* anger-hostility, *C-B* confusion-bewilderment, *D-D* depression-dejection, *F* friendship, *F-I* fatigue-languid, *GI* gastrointestinal, *POMS* profile of mood states, *T-A* tension-anxiety, *TMD* total mood distress, *V-V* vigor-vitality. Data represent the means ± standard deviation (SD). *P*-value is based on the Mann-Whitney U-test. Significance is at the 5% level

The VAS of anxiety for upper GI endoscopy post-distraction was significantly lower than that pre-distraction in group 2 (*p* <  0.05). The VAS of strain for upper GI endoscopy post-distraction was significantly lower than that pre-distraction in group 3 (*p* <  0.01). The VAS of strain, anxiety, and fear for upper GI endoscopy post-distraction was significantly lower than that pre-distraction in group 4 (*p* <  0.001, *p* <  0.05, and *p* <  0.05).

### Impression of distraction after upper GI endoscopy

The comparison of the impression of distraction after upper GI endoscopy among the 3 distraction groups is shown in Table 5. The VAS of satisfaction for distraction in group 2, group 3, and group 4 was 62.7 ± 17.7, 63.4 ± 16.9, and 72.6 ± 19.1, respectively. There was a significant difference in the satisfaction for distraction among the 3 distraction groups on the Kruskal Wallis-test (< 0.01). In post-hoc pairwise comparisons, the satisfaction for distraction in group 4 was significantly higher than that in group 2 and group 3. The VAS of willingness for the use of distraction at next examination in group 2, group 3, and group 4 was 71.9 ± 16.6, 72.4 ± 20.2, and 76.4 ± 18.3, respectively, and although there was no significant difference among the 3 distraction groups, the VAS in all distraction groups was excellent in comparison.

**Table 5:** Comparison of the impression of distraction after upper GI endoscopy among the three distraction groups

	Group 2	Group 3	Group 4	*P*-value
Satisfaction for the distraction	62.7 ± 17.7^a^	63.4 ± 16.9^a^	72.6 ± 19.1^b^	< 0.01
Willingness for the use of distraction	71.9 ± 16.6	72.4 ± 20.2	76.4 ± 18.3	NS

*GI*, gastrointestinal

Data represent the means ± standard deviation (SD)

*P*-value is based on the Kruskal Wallis-test. Significance is at the 5% level. Post hoc pairwise comparison were conducted by Mann-Whitney U test with the Bonferroni correction; different letters indicated a significant difference at the 0.01667 (0.05/3) level

## Discussion

The aim of the present study was to investigate the influence of interventions using audio distractions, visual distractions, and the combination of auditive and visual distractions for a person who is going to undergo upper GI endoscopy soon. Although there have been several studies on auditive and visual effects using subjective assessments such as anxiety and satisfaction during various endoscopies [1, 3, 15–21], there have been few reports on the influence of distraction for a person who is going to undergo upper GI endoscopy soon using objective assessments such as autonomic nervous function. The present study demonstrated that, although auditive distraction alone and visual distraction alone improved psychological evaluation, cardiovascular responses, and some autonomic nervous function parameters prior to upper GI endoscopy, the combination of auditive and visual distractions may be more effective. 

It has been reported that several distraction techniques based on visual, auditory, and olfactory stimulation were effective for the reduction of pain and anxiety during various medical inspections, care procedures, and treatments. In particular, music therapy has been used in a range of healthcare settings, including oncology, dementia, palliative care, and hospices [22, 23]. Recently, the positive effect of music for various different endoscopic procedures has been reported in several studies [1, 15, 24–29]. Kotwal MR et al. reported that there was a statistically significant effect of music on blood pressure and respiratory rate during gastroscopy between patients with and without music [24]. Bampton et al. reported that there was no significant difference in the overall tolerance score between the music group and the no-music group; however, a significantly higher proportion of patients described the experience of the GI endoscopic procedure as being at least moderately unpleasant in the no-music group [25]. The number of positive effect articles of music on anxiety levels for upper GI endoscopy may be slightly more than that of negative effect articles. The present study showed that the score in 4 items of POMS, blood pressure, and the LF power/HF power ratio immediately pre-upper GI endoscopy were significantly lower than that at baseline conditions in the music group.

Distraction due to visual stimulation has been used for various medical procedures [8, 30–32]. For example, visual stimulation using video glasses showed a hypoalgesic effect for experimental pain in a cold pressor test [30]. Also, there have been several studies on the influence of visual stimulation on colonoscopy. Umezawa et al. showed that there was no significant difference in the median anxiety score, median pain score, and SBP before, during, and after colonoscopy between the 2 groups watching a silent movie using a head-mounted display or only wearing the display; however, the median post-procedural satisfaction levels and the rate of wishing to use the same method for the next procedures in the subjects watching the silent movie was significantly higher than that in the subjects only wearing the display [32]. On the other hand, Lee DW et al. demonstrated that there was no significant difference in the dose of propofol for sedation and pain scores between 52 subjects with visual distraction and 53 subjects without visual distraction during colonoscopy [8]. The influence of visual distraction on colonoscopy has remained controversial, and to our knowledge, there has been no study of the influence of visual distraction for upper GI endoscopy. The present study showed that the scores of 5 items of POMS and strain level for upper GI endoscopy immediately pre-upper GI endoscopy were significantly lower than that at baseline conditions in the visual group. Additionally, blood pressure and the LF power/HF power ratio immediately pre-upper GI endoscopy were significantly lower than that at baseline conditions in the visual group.

To our knowledge, there have been 2 prospective randomized controlled trial studies investigating the influence of the combination of auditive and visual distractions on intestinal endoscopy examination [7, 8]. Lee DW et al. compared the influence of the combination of auditive and visual distractions with the influence of a visual distraction alone for sigmoidoscopy [8]. This study showed that the dose of propofol for sedation of sigmoidoscopy in 52 subjects with a combination of auditive and visual distractions was significantly lower than for 52 subjects with a visual distraction alone. However, there was no significant difference in the pain score, the satisfaction score, and the rate of willingness to repeat the same procedure for next examination between the 2 groups. On the other hand, Lembo et al. showed that the level of discomfort and anxiety during a flexible sigmoidoscopy in subjects with the combination of auditive and visual distractions group was lower than in the other 2 groups (no intervention group and audio distraction group) [7]. The present study showed that the score in 5 items of POMS, the impression for upper GI endoscopy, vital signs, and the LF power/HF power ratio immediately pre-upper GI endoscopy were significantly lower than that at baseline conditions in the combination group. Additionally, the decrease in the rate of P and DBP was significantly higher than in the other 3 groups. Also, the satisfaction for the distraction after upper GI endoscopy was highest in all distraction groups. The present study showed that the combination of auditive and visual distractions creates the most positive effect of all the distractions. Distraction may reduce anxiety by limiting attentional capacity, namely drawing attention away from upper GI endoscopy and induce an improvement of vital signs and psychological factors by stabilization of the balance of autonomic nervous function.

The present study had some limitations that should be noted. First, there was a possibility of different results between persons that undergo upper GI endoscopy for the first time and those repeating the upper GI endoscopy because the mean number of upper GI endoscopy in all subjects of the present study was 4 times. Further investigation of subjects who undergo upper GI endoscopy for the first time or comparison between persons who undergo upper GI endoscopy for the first time and repeat patients will be required. Second, there was the possibility of a selection bias, because all of the participants in the present study were healthy individuals who hoped to undergo a medical check-up. It is unclear whether results in sick persons or an elderly population would produce similar results to the present study. Further studies will be necessary to resolve these limitations.

## Conclusions

The present study demonstrated that auditive distraction and visual distraction were effective, however, a combination of auditive and visual distraction was more effective than any other group according to subjective and objective appropriate evaluations. Although it is important for persons receiving upper GI endoscopy to discover GI lesions, it should be considered necessary to improve various physical and psychological conditions before upper GI endoscopy. The distractions in the present study, which were simple, safe, and low-cost, may play an important role in improving physical and psychological factors before upper GI endoscopy.

## Abbreviations

DBP: Diastolic blood pressure
GI: Gastrointestinal
HF: High-frequency
HRV: Heart rate variability
LF: Low-frequency
P: Pulse
POMS: Profile of mood states
PSA: Power spectral analysis
SBP: Systolic blood pressure
SpO_2_: Peripheral blood oxygen saturation
VAS: Visual analog scale
